# Explainable AI for Data-Driven Feedback and Intelligent Action Recommendations to Support Students Self-Regulation

**DOI:** 10.3389/frai.2021.723447

**Published:** 2021-11-12

**Authors:** Muhammad Afzaal, Jalal Nouri, Aayesha Zia, Panagiotis Papapetrou, Uno Fors, Yongchao Wu, Xiu Li, Rebecka Weegar

**Affiliations:** Department of Computer and Systems Sciences, Stockholm University, Stockholm, Sweden

**Keywords:** self-regulated learning, recommender system, automatic data-driven feedback, explainable machine learning-based approach, dashboard, learning analytics, AI

## Abstract

Formative feedback has long been recognised as an effective tool for student learning, and researchers have investigated the subject for decades. However, the actual implementation of formative feedback practices is associated with significant challenges because it is highly time-consuming for teachers to analyse students’ behaviours and to formulate and deliver effective feedback and action recommendations to support students’ regulation of learning. This paper proposes a novel approach that employs learning analytics techniques combined with explainable machine learning to provide automatic and intelligent feedback and action recommendations that support student’s self-regulation in a data-driven manner, aiming to improve their performance in courses. Prior studies within the field of learning analytics have predicted students’ performance and have used the prediction status as feedback without explaining the reasons behind the prediction. Our proposed method, which has been developed based on LMS data from a university course, extends this approach by explaining the root causes of the predictions and by automatically providing data-driven intelligent recommendations for action. Based on the proposed explainable machine learning-based approach, a dashboard that provides data-driven feedback and intelligent course action recommendations to students is developed, tested and evaluated. Based on such an evaluation, we identify and discuss the utility and limitations of the developed dashboard. According to the findings of the conducted evaluation, the dashboard improved students’ learning outcomes, assisted them in self-regulation and had a positive effect on their motivation.

## 1 Introduction

Providing feedback is one of the many tasks that teachers perform to guide students towards increased learning and performance, and it is viewed as one of the most powerful practices to enhance student learning (Henderson et al., 2019). However, when there are many students in a class, as is often the case in higher education, it becomes very challenging for teachers to provide all students with effective guidance individually. Moreover, it is challenging for teachers to understand which course activities and resources should be delivered as action recommendations that could help student to improve their learning and performance (Winstone and Carless 2019). It has also been shown that effective feedback and action recommendations are essential for self-regulated learning (SRL) and are significantly correlated with students’ learning and performance (Algayres and Triantafyllou 2020). Therefore, to address these challenges, a number of studies within the fields of learning analytics (LA), artificial intelligence in education (AIED) and educational data mining (EDM) have investigated how students’ self-regulation could be supported through, for instance, dashboards that provide predictive student performance (Lakkaraju et al., 2015; Johnson et al., 2015; Kim et al., 2016; Marbouti et al., 2016; Akhtar et al., 2017; Chanlekha and Niramitranon 2018; Choi et al., 2018; Howard et al., 2018; Nguyen et al., 2018; Predić et al., 2018; Villamañe et al., 2018; Xie et al., 2018; Baneres et al., 2019; Bennion et al., 2019; Rosenthal et al., 2019; Nouri et al., 2019; D.). These studies employed various data mining, machine learning (ML), clustering and visualisation techniques on a diverse variety of learning management system (LMS) data sources to predict student success and failure in a course or in an entire academic year. In these studies, the obtained prediction outcomes were used as feedback for the students. Although such feedback might be helpful to some extent, it does not provide any insightful information and actionable recommendations that could help students reach their desired academic performance (Baneres et al., 2019).

Nevertheless, some studies have moved beyond the presentation of prediction results and extracted pivotal factors that could affect students’ performance over time and utilised those factors as recommendations (Akhtar et al., 2017; Lu et al., 2018; Ahuja et al., 2019; Bibi et al., 2019; Kamal and Ahuja 2019). For instance, Lu et al. (2018) performed regression analyses, and Bibi et al. (2019) employed neural networks in order to identify specific factors that influence students’ performance and recommendations to improve those factors. However, these factor-identifying approaches are not helpful for students to improve their learning behaviour, as most identified factors are non-changeable (non-actionable knowledge) or are not related to the course work. Examples of this are factors such as family support, marks in the previous degree, and current cumulative grade point average (CGPA). Furthermore, the current approaches do not provide students with actual explanations of the predictions and do not utilise dashboards that provide automatic and intelligent guidance in the form of recommendations to students during ongoing courses, such as recommendations that, for instance, guide students towards the learning material or activities that will increase the probability for increased course performance. Such explanations and dashboards would help students to regulate their behaviour in a data-driven manner. Moreover, the existing approaches have overlooked the prediction and guidance regarding student performance at the assignment or quiz level in courses currently running (Kuzilek et al., 2015).

Against this background, this study addresses the following research questions:

1) Can we employ explainable ML approaches to identify factors that affect student academic performance at the assignment or quiz level in order to provide automatic and data-driven feedback?
2) What is the utility and limitations of a dashboard designed to provide automatic data-driven feedback and action recommendations to students based on the above explainable ML approach?

This paper aims to answer the questions stated above. Hence, in this paper, we propose an explainable ML-based approach that first predicts student performance on each assignment and quiz using ML algorithms. Secondly, we identify the performance-influencing factors based on each prediction by employing explainable ML techniques on the built predictive models. Lastly, automatic and intelligent feedback is computed using the predictive student performance and identified factors for students. The main contribution is that we combine a prediction approach with an explainable ML approach that, in comparison with previous studies, allows for fine-grained insights that support the provision of detailed data-driven actionable feedback to students, which explains the “why” of the predictions. That is, we present an approach that gives students more actionable information than what can be achieved through just informing them about the prediction.

In this paper, we develop a data-driven feedback and intelligent action recommendation (DFIAR) dashboard based on the proposed explainable ML-based approach. To evaluate the utility and limitations of the developed dashboard, two evaluations were conducted in two different settings; firstly 1) an experts’ workshop evaluation was conducted to obtain feedback on the developed dashboard, and secondly, 2) a dashboard was developed and tested in a real educational setting (university course) in order to evaluate the dashboard’s utility and limitations.

The remainder of this paper consists of five sections. In *Related Work*, an overview of the existing approaches is given. In *Methods*, our approach is presented. In *Results*, the results obtained from applying the proposed approach are presented. *Discussion* discusses the results, possible directions for future research and the limitations of the study. In *Conclusion*, all the findings reported in this study are summarised.

## 2 Related Work

This section presents the related work in AIED, EDM and LA research streams of the education field, which have been proposed to predict student academic performance and the identification of factors that affect their performance. The objective of this section is to analyse recently proposed approaches and to identify their limitations and drawbacks. A closer comparison with relevant work from the AIED, EDM and LA literature is presented in the following sections.

### 2.1 Student Academic Performance Prediction

Existing studies under AIED have proposed several ML frameworks (Johnson et al., 2015; Howard et al., 2018; Xie et al., 2018; Bennion et al., 2019; Rosenthal et al., 2019; Xu et al., 2019; Rivas et al., 2021) to develop early warning systems (EWS) that could offer early prediction of at-risk students. In terms of data utilisation, most of the frameworks have collected previous academic performance and LMS usage of graduate students, but some have focused on data of medical students (Bennion et al., 2019; Rosenthal et al., 2019). On the other hand, Johnson et al. (2015) worked on a broader scale by analysing four districts’ school data. After collecting data, the proposed frameworks extracted various features, such as online study duration, frequency of internet connectivity and document and video views to train ML algorithms (neural networks, tree-based methods and logistic regression) to predict at-risk students, and they gained up to 90% accuracy. Based on prediction results, the proposed frameworks identified that around 50% of students had similar patterns when they were at risk. At the same time, the frequency of internet connectivity and access to the learning material proved a direct relation with the risk of student failure. In implementing EWS, Howard et al. (2018) found that five to 6 weeks was an optimal time to intervene during the course.

Studies under EDM proposed several data mining approaches (Chanlekha and Niramitranon 2018; Kaviyarasi and Balasubramanian 2018; Predić et al., 2018; Zaffar et al., 2018; Fernandes et al., 2019; Inyang et al., 2019; Li et al., 2019) to predict academic performance. In contrast to AIED, specific subject-related data were collected (e.g. engineering, computer graphics, mathematics, etc.). However, Fernandes et al. (2019) collected entire school data to forecast students’ academic performance. Chanlekha and Niramitranon (2018) aimed to develop EWS for at-risk students in the traditional information-lacking classroom environment. Similar to AIED, students’ previous academic performance, demographics and LMS usage-related features were extracted; however, as compared to AIED, most of the studies employed ensemble association rules and clustering to identify a student who would pass the course or not. For instance, Predić et al. (2018) developed a voting tool based on several ML algorithms and determined student status based on the number of votes from each algorithm, obtaining 90% accuracy. On the other hand, Inyang et al. (2019) employed hierarchical cluster analysis to classify students into four groups (dropout, spillover, high achiever and low achiever).

Studies under LA proposed diverse approaches (Marbouti et al., 2016; Adadi and Berrada 2018; Choi et al., 2018; Nguyen et al., 2018; Villamañe et al., 2018; Baneres et al., 2019; Nouri et al., 2019) to predict academic performance and provide proactive guidance to both students and teachers. Although both EDM and LA research streams shared compatible goals, the subtle differences were their ontological origins, techniques used and even research questions. Regarding data utilisation, a few approaches (Marbouti et al., 2016; Dominguez et al., 2018; Villamañe et al., 2018) have utilised extensive data from online engineering or business courses. In contrast, data were collected from a small number of students enrolled in blended learning courses using other approaches (Akhtar et al., 2017; Deng et al., 2019; Nouri et al., 2019). Therefore, assessment grades, demographic and LMS-based clicker features were extracted from online course data; however, in blended learning courses, alongside the quiz scores other focusing features were log data and student interactions with teachers and peers (Nguyen et al., 2018). For online learning courses, features were used to model different ML algorithms (KNN, Naïve Bayes, support vector machine, k-nearest neighbours and decision tree) and achieved a higher accuracy of 85% (Marbouti et al., 2016). For blended learning features, regression (for predicting the number of interactions), clustering (for creating a cluster of low-, medium- and high-level at-risk students), and classification (to categorise students into A–F grades) techniques were employed and obtained 75% correct predictions.

Several studies (Akhtar et al., 2017; Dominguez et al., 2018; Deng et al., 2019; Hlosta et al., 2019) proposed visualisation approaches for various purposes, such as categorisation of student profiles, visualisation of key milestones and creation of a student academic performance dashboard. In terms of data utilisation, Villamañe et al. (2018) collected heterogeneous data related to assessment grades from online and physical classroom settings, while Dominguez et al. (2018) gathered data from higher education institutions across Europe. A couple of studies (Akhtar et al., 2017; Deng et al., 2019) focused on course-specific data features, such as obtained exam scores and question structure. In these works, t-SNE (t-distributed stochastic neighbour embedding), Pearson correlation and linear regression were applied to determine the significance of analysing educational attributes. For evaluation, Villamañe et al. (2018) used COBLE (competence-based learning environment) that completely satisfied 62.5% of lecturers, and in Deng et al. (2019), the PerformanceVis tool of visual data analytics was applied and demonstrated its effectiveness. Furthermore, a coordinated view of data as hypercubes was useful for prototype implementation that leverages coordinated histograms along with interactive dimensionality reduction and statistical tests, t-tests and Wilcoxon rank-sum tests, that improved the student retention rate by 49% (Hlosta et al., 2019).

However, existing approaches do not provide an explanation (feedback) for “why” a student would possibly fail or pass a course, which could be helpful for students to improve their performance. These approaches only provide the outcomes of the predictions as feedback for both teachers and students. Although such feedback might be helpful to some extent, it does not provide any meaningful insights or actionable information about the reasons behind the prediction—that is, students and teachers do not receive actionable feedback. Moreover, existing approaches have overlooked the prediction of student academic performance at the assignment or quiz level in current courses. Such information can be helpful for teachers who are planning interventions or other strategies to improve the student retention rate (Kuzilek et al., 2015). Furthermore, most of the models for identifying the success or failure of students were trained on the data related to the results of their previous courses instead of the course currently running. That could cause inaccurate predictions related to student performance in the current course because some courses are completely different from others.

### 2.2 Identification of Factors That Influence Students Performance

Considering that predictive models are not able to identify the factors that influence student academic performance, several studies (Akhtar et al., 2017; Lu et al., 2018; Ahuja et al., 2019; Bibi et al., 2019; Kamal and Ahuja 2019) have extracted the pivotal factors that could affect student learning outcomes over time. Lu et al. (2018) performed regression analyses on student data and found four online (e.g., activities per week, video play clicks, videos backwards seek clicks and weekly practice scores) and three traditional (e.g., homework score, quiz score and participation in after-school tutoring) factors that affected student academic performance, while Bibi et al. (2019) employed a neural network on four semesters worth of data and found that CGPA, student competence in English, learning a skill, marks in the previous degree, family support, social media, and teacher role significantly affect the academic performance of students.

In another context, (Kamal and Ahuja 2019), created a questionnaire containing 80 questions and employed a decision tree and a regression approach to identify the influential factors. The approach found that attendance, estimated chance of academic success, secondary stream opted and study skills are significant factors that affect academic performance. Similarly, Ahuja et al. (2019) employed both decision tree and clustering methods to identify the factors influencing enhanced online learning and found that the performance of low-performing students could improve by making them active in online discussions. On the other hand, during collaborative and self-regulated learning, seven factors were found to be essential, namely attendance, time spent in class, sitting position, sitting in groups for collaborative learning and self-efficacy, positive strategy use, feeling less anxious and less harmful strategy use for self-regulated learning (Akhtar et al., 2017; Pardo et al., 2019). Koedinger et al. (2015) identified the factors causing students to drop out for MOOC users, open learning initiative (OLI) users and MOOC + OLI users. The identified factors, such as participation rate in attempting quizzes and achieved quiz scores, yielded the lowest dropout of MOOC + OLI users. Moreover, the impact of course features (reading, writing and doing) on learning was determined, and their in-between causal relationships were derived. The results obtained by the experiments revealed that the students who did more practical activities, regardless of reading and watching, were high achievers on during-course quizzes. However, on the final exam, watching videos or reading content were significantly important, as were practical activities. However, some features, like forum discussion and peer assignments, which are considered essential factors for student learning, were ignored in this work.

Nevertheless, the available approaches are not helpful for students to improve their learning behaviour because most identified factors are non-changeable (non-actionable knowledge) or are not related to the course. For example, factors such as family support, marks in the previous degree, and current CGPA could be the reason behind a decline in student performance. However, it is not possible for students to change or improve these factors during the course. These factors are also less helpful for teachers, as the teachers have limited influence over them, and they inform less how to restructure a course or provide additional resources to students.

### 2.3 Self-Regulation Learning

The theory of Self-regulated learning (SRL) is a mechanism that supports students by providing them control over their learning process (Manso-Vázquez and Llamas-Nistal, 2015). It is expected that students have knowledge about their current learning status and are well-informed about applying the cognitive strategies for assisting them in reaching the anticipated goals of learning (Capuano et al., 2012). In the existing literature, numerous studies focused on the benefits of SRL in students’ academic performance by making their learning process more autonomous (Broadbent and Poon 2015; Kizilcec et al., 2017; Fincham et al., 2018; Rienties et al., 2019). To support this claim, Hattie (2008) conducted a meta-analysis of 800 metastudies. Results demonstrated that the desired goal of successful learning could be achieved by applying SRL strategies.

However, prior studies reported that in online learning, students struggle with self-regulation (Kizilcec et al., 2017). In other words, while taking online courses, students are needed to self-monitor themselves to complete a course (Sambe et al., 2017). It becomes incredibly challenging for students with limited skills of SRL. Kopeinik et al. (2014) argued that online learning platforms such as Moodle usually ignore individual students’ needs. Although some teachers attempt to improve learning design by incorporating SRL elements, the lack of timely and personalized feedback to students reduces the effectiveness of SRL. This leads to students feeling discouraged although they follow the SRL strategies designed by their teachers (Kizilcec et al., 2016). Eventually, the deficiencies in SRL and the reception of improper guidance from teachers result in the reduction of effectiveness of the self-regulation and the overall goal pursuit (Shih et al., 2010).

These challenges endorse the need for SRL among online students to be aware of their learning status and guide them in improving their learning during a course. In the recent past, there has been an increasing tendency to use data analytics to measure self-regulation. Some learning platforms provide essential information as a learning analytics dashboard by tracking students’ online data (ElSayed et al., 2019). Compared to traditional measures, learning analytics and data mining are becoming prevalent as these are relatively more convenient and accurate. However, such learning analytics and data mining-based dashboards do not provide guidance through automatic and intelligent feedback and action recommendations (for instance, guide students towards the learning material or activities that will increase the probability for increased course performance) as well as not provide students with actual explanations of the predictions during ongoing courses. Such explanations and dashboards would help students to regulate their behaviour in a data-driven manner.

## 3 Methods

**FIGURE 1:**
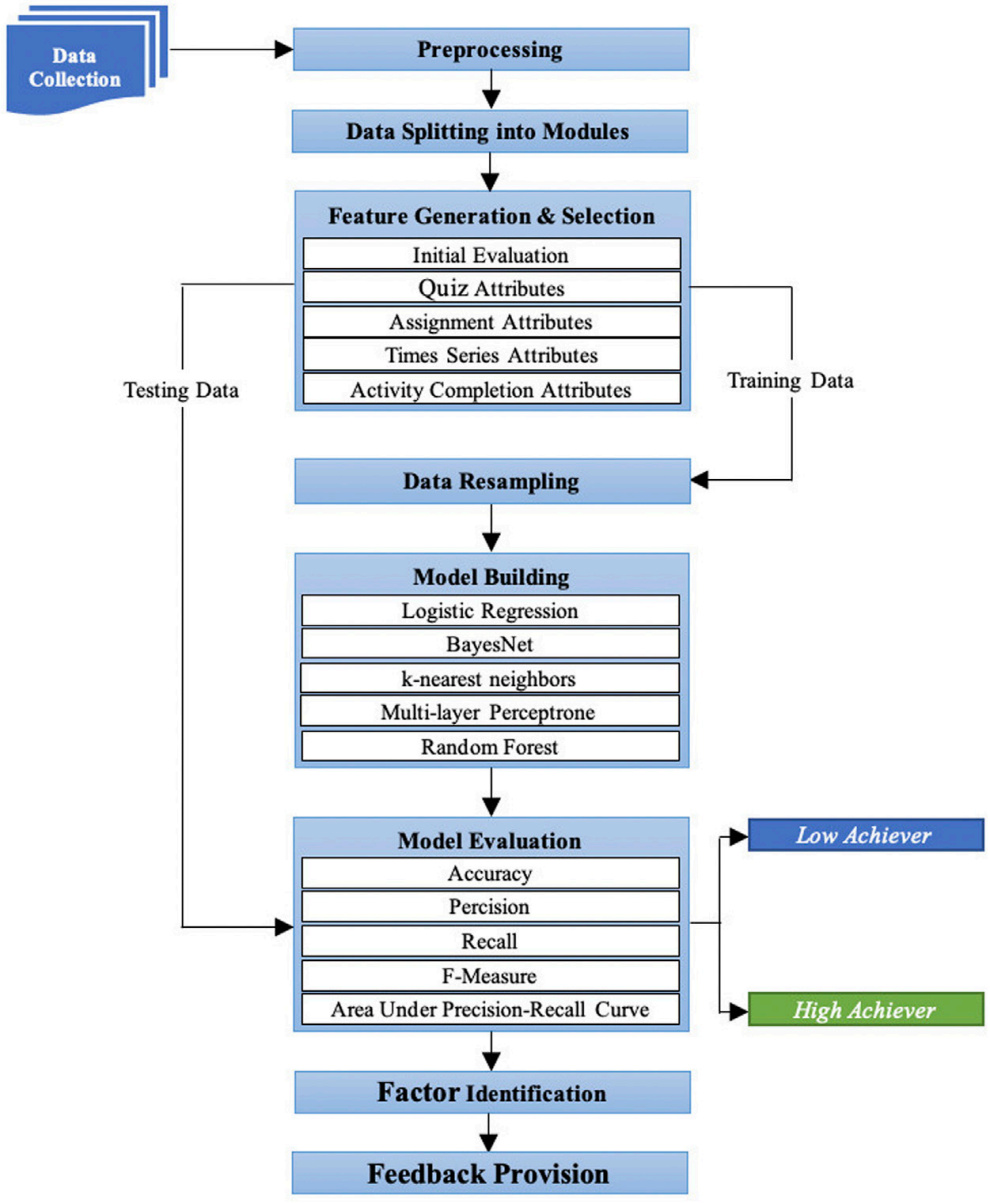
An explainable machine learning-based approach to predict and explain student performance at the assignment level.

In this section, an explainable ML-based approach is presented that predicts student performance at the assignment level, identifies the influencing factors that affect student performance, and computes informative feedback for both students and teachers to provide meaningful insight throughout the course. Figure 1 depicts the main phases of the proposed approach. First, student data about different social and educational activities were collected from the LMS. Second, preprocessing was performed on the collected data to link students and their activities and remove irrelevant data. Third, preprocessed data was split into modules since the proposed approach provided guidance at an assignment or quiz level. Fourth, features were generated from each module and a feature selection strategy was employed on the generated features to remove irrelevant features and to improve the accuracy of the predictive model. Fifth, data were resampled to avoid the class imbalance problem before model building. Sixth, predictive models were built based on the features generated using established ML algorithms. Seventh, the built predictive models were evaluated using advanced evaluation measures [accuracy, precision, recall, F-measure and area under the precision-recall curve (PRC)], and the best one was selected using a cross-validation procedure. Eight, the best predictive model selected was used with explainable ML techniques to identify the influential factors that created hurdles for students to reach their desired performance. Ninth, automatic and intelligent feedback was computed for students based on students’ performance predictions and identified factors in a usable and actionable way that helps them to regulate their behaviour in a data-driven manner. Lastly, a dashboard was designed and evaluated under different settings to examine the utility and limitations of the dashboard.

### 3.1 Data Collection

The data in this study were from a programming course that was taught consecutively for 2 years (2019 and 2020) to distance-learning students using an LMS at Stockholm University, Sweden. During 2019, 82 students enrolled in the course, and out of those, 61 students were active during the course, while 21 students did not log in to the LMS after registration. During 2020, 134 students enrolled in the course, and this year, 96 students were active during the course, whereas 38 students did not log in to the LMS after registering for the course. We asked the students to give consent for research at the beginning of the course through an online survey. Hence, the inclusion criteria were that the students had to attempt quizzes and assignments, and they had to actively participate in the course activities, watch video lectures, and study reading material in the LMS. Based on these criteria, 59 students were excluded from the data, and 157 students were selected for this study. Table 1 presents a summary of the data set which was chosen for this study. After selecting the students, course data were extracted from the LMS in the form of JSON files.

**TABLE 1:** Data set summary.

Year	Enrolled students	Selected students
2019	82	61
2020	134	96
2019–2020	216	157

### 3.2 Preprocessing

After collecting the data, preprocessing was performed on the downloaded data files to link students with their activities (educational and social), remove data redundancy, maintain the students’ anonymity and for student labelling. The preprocessing phase consisted of four steps. First, to keep students anonymous, we did not use student names directly but a unique identifier (ID) that we generated randomly and replaced with student names in all data files. Second, an indexing structure was created that stored the relationship between student IDs and their activities, which provided the opportunity to extract student activities at once using their IDs. In the third step of preprocessing, we removed the incomplete and duplicate quiz or assignment attempts. Last, each student was labelled according to their performance on different assessment activities (quizzes and assignments). The criteria were if a student got below or equal to 75% on an assessment activity, they were considered a low achiever, and if the student got above 75%, they were considered a high achiever.

### 3.3 Data Splitting Into Modules

**FIGURE 2:**
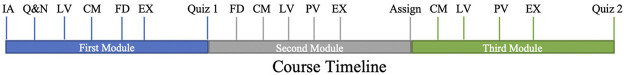
Course timeline, where IA represents initial assessment, Q and A represents questions and answers, LV represents lecture videos, CM represents course reading materials, FD represents forum discussion, PV represents practical videos, Assign represents assignment, and EX represents programming exercises.

Since the proposed approach provides a prediction at an assignment or quiz level, the data were split into multiple modules. The rationale behind this split is that instead of dividing data weekly, we divided the course into three different modules, as shown in Figure 2. Each module is associated with each other module, where the first module is theoretical and is based on video material, reading content and a primary programming knowledge assessment activity (quiz 1: basic questions about various programming concepts, such as loop, event and function). The second module comprises scratch programming consisting of video guides and a practical assignment (make a simple game using block programming). The third module is the *Python* module, where the students do many exercises guided by videos, followed by an examination task (quiz 2: advanced questions about programming concepts, selecting an appropriate solution for problems, finding errors from code). Hence, three modules were created from the preprocessed data set. Figure 2 is a graphical representation of the split modules.

### 3.4 Feature Generation

After splitting the data into modules, features that could be categorised into five categories based on their performance were generated: 1) an initial assessment contained information about student experiences in programming, tool utilisation and motivation to collect initial assessment information. A survey that had questions about previous experience and motivation was conducted before the beginning of the course; 2) quiz attributes contained each quiz score (1 for a correct answer and 0 for incorrect), total quiz score, number of attempts, and time spent on the quiz; 3) assignment attributes contained the grade and the number of days needed to complete the assignment; 4) activity completion attributes contained the number of views for each video, the number of course material views and the number of discussion forum views; and 5) time-series-based attributes had information about the LMS usage behaviour of each student. The process to generate these attributes from LMS usage data consisted of four steps. First, the number of clicks for each student obtained in the period of the selected module (30 days for each module) was collected to generate their respective time series. Second, Discrete Fourier Transform (DFT) was applied to the generated time series that provided a periodogram. Third, a set of 20 most intensive DFT coefficients was collected from the periodogram of each student. Those sets of 20 coefficients were not identical for all students. Therefore, a new list was formed containing coefficients that appear in all sets. It led to creating a table in which list coefficients were specified in the first column and their intensities for each student in other columns. Afterwards, the table was sorted to get coefficients more frequently occurring among all students. At last, the coefficients were reduced by eliminating the least represented ones and these eventually obtained coefficients were used as time-series based attributes (Gamulin et al., 2016).

### 3.5 Feature Selection

The next phase of the proposed approach was feature selection, during which relevant and significantly important features were selected from the generated feature since the predictive models build on several features, many of which do not have any association with student performance. Therefore, to remove the irrelevant features, a feature selection strategy was introduced in this study.

The procedure of the employed feature selection strategy, as given in Algorithm 1, was mainly a two-step process in which information gain (InfoGain) (Stachniss et al., 2005) computation was first performed and the analysis of variance (ANOVA) (Cuevas et al., 2004) statistical test was then applied. In this algorithm, input included the set of features generated in *Feature Generation* ( $F_{e}\left[\right]$ ), a threshold value for ranking the features (ranker_threshold ) according to their importance and the critical F-value ( $F\_value_{\propto =0.05}$ ) for the ANOVA test, as shown in line 1. The output provided a set of the most positively discriminating features selected from the generated features given by line 2. In initialisation, ranker_threshold was assigned the value of 0.5, as provided by line 3. According to this strategy, in the first step, each feature in the generated features ( $F_{e}\left[\right]$ ) was taken one by one in the *for* loop shown in line 4.

**Algorithm 1:** Feature Selection Strategy

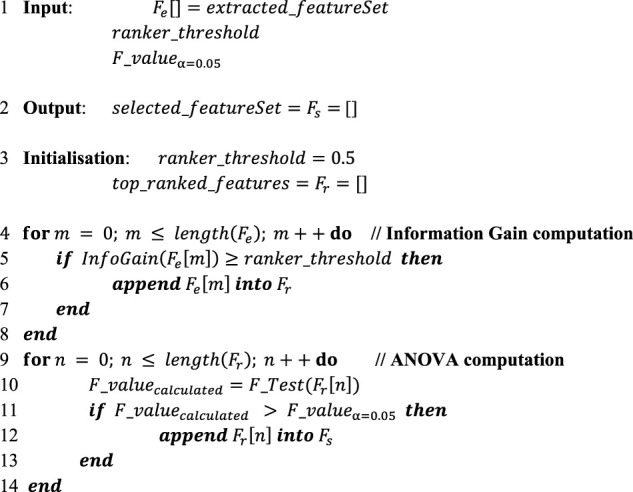

The InfoGain was computed for each feature, and its value was compared with the predefined value of ranker_threshold by using the *if* condition in line 5. A particular feature that had an InfoGain greater than or equal to 0.5 was added to the list of top-ranked features ( $F_{r}\left[\right]$ ), as in line 6. In the second step of the ANOVA calculation, the statistical F-test was applied to each of the obtained top-ranked features to obtain the F-value ( $F\_value_{calculated}$ ), indicating the feature’s significance based on the variance analysis given in lines 9 and 10. The calculated F-value was compared with the critical value ( $F\_value_{\text{α}=0.05}$ ) on the F-distribution table of error ( α ) 0.05. If the feature-calculated F-value was greater than the critical value, this feature was added to the selected features ( $F_{s}$ ) shown in lines 11–14.

### 3.6 Data Resampling

One of the most common ML problems is a class imbalance, where the total number of instances in a class of data is far less than the total number of instances in another class of data. In programming course data, we had the same problem because the number of high-achieving students was smaller than the number of low-achieving students. Therefore, resampling was performed on the data to ensure that almost the same number of instances from all classes existed in the given data set. The ultimate objective was to remove the class overfitting issue that appeared due to class imbalance data. In this work, the synthetic minority oversampling technique (SMOTE) (Chawla et al., 2002) was employed for data resampling, which randomly increases minority class instances by replicating them. Eventually, it outputs data with a balanced class distribution. For example, in any course, the number of students who achieve a lower grade on an assignment is less than those who earn a higher grade. The application of SMOTE generates more instances of lower-grade students to equalise that of higher-grade students. The class distribution for each module is provided in *Prediction of Second Module Assignment Performance* and Third Module Quiz Prediction below.

### 3.7 Model Building and Evaluation

In this study, we employed six predominant ML algorithms for performance prediction, namely logistic regression (LR), k-nearest neighbours (KNN), support vector machine (SVM), random forest (RF), multi-layer perceptron (MLP), and BayesNet. The purpose was to compare and evaluate these classifiers thoroughly to determine which one consistently performs better. To ensure that the built model generalises well, the predictive model’s performance was evaluated using accuracy, precision, recall, F-measure and area under the PRC. In the experimental set-up, a nested cross-validation (Wainer and Cawley 2021) procedure was applied where ten folds were created, and we ensured that only training data were balanced using SMOTE. Moreover, students that were in the training set were not in the test set and vice versa.

### 3.8 Factor Identification Through an Explainable Machine Learning-Based Algorithm

By only predicting students’ performance, neither students nor teachers would be made aware of why a student would achieve a low or high performance on the next assignment or quiz. Consequently, students could not self-regulate, and teachers could not provide adequate course resources to enhance students’ performance. Thus, in this study, we proposed an explainable ML-based algorithm, as indicated by Algorithm 2, to identify influencing factors supporting students’ self-regulation and teachers in improving course structure and content. The goal of the proposed algorithm was that if a student was predicted to achieve a lower grade then the algorithm would identify factors that would affect student performance and later assist the student in how the desired grade could be achieved. This information could be helpful for teachers to improve course structure and resources.

**FIGURE 3:**
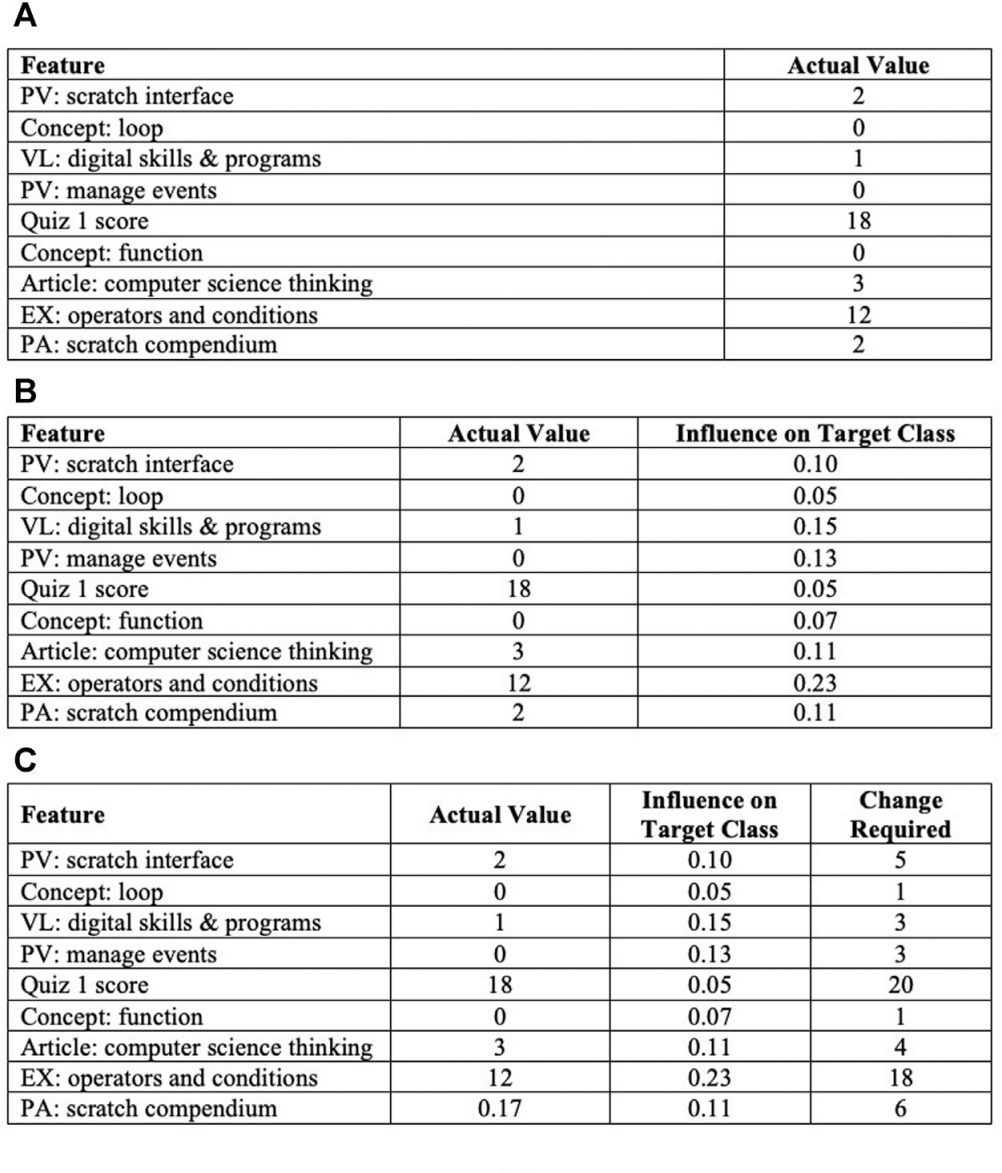
The output of Algorithm 2, which shows the extracted influence value of each factor and change required to meet the desired performance.

The workflow of the proposed algorithm (Algorithm 2) is as follows: it took instance (student) features and the actual values of these features [as shown in Figure 3 (a)], training set, target class (high achiever if a student was predicted to be a low achiever), predictive model and library of explainable model-agnostic explanations (LIME) (Ribeiro et al., 2016) as the input given by line 1, and it provided potential factors as output as shown in line 2. The initialisation step in line three specified the feature impact threshold as 20. Under this set-up, first an explanation was generated by passing the built model, instance features and feature impact threshold to the LIME as arguments. Intuitively, a generated explanation is a local linear approximation of the predictive model’s behaviour. While the model may be very complex globally, it is easier to approximate it around the vicinity of a particular instance (Ribeiro et al., 2016).

**Algorithm 2:** Factor Identification Using Explainable ML

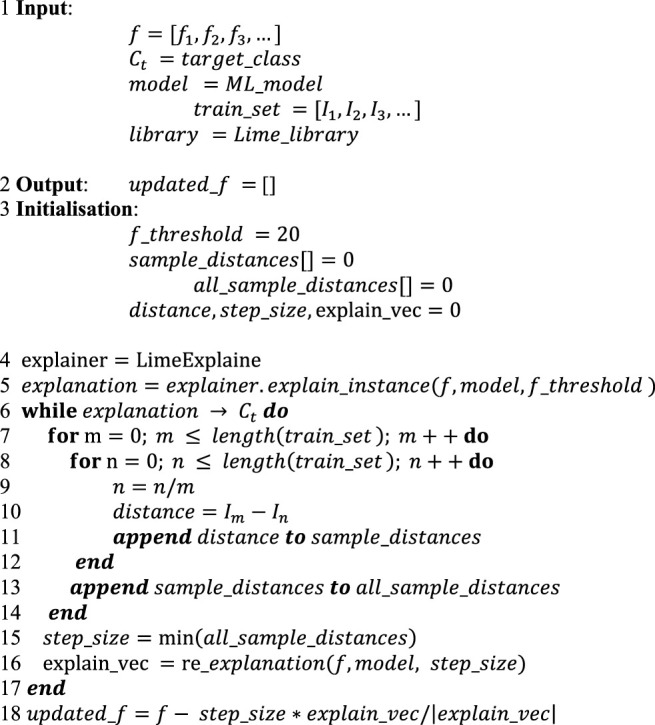

The explanation provides the influence value of each feature on the target class for individual students as shown in Figure 3B. Since few features did not have a substantial influence, a threshold (20) was applied to extract the highest influence features, as shown in line 5. Here we have features that influenced the prediction, but how much change in features would be sufficient was not explained. For example, one explanation found that the EX: operators and conditions feature had a high influence on a student’s upcoming task (e.g. assignment) performance. The student needed to improve their score before attempting the assignment, but how much would be enough was not provided. Therefore, in the third step, the obtained explanation feature vector was calculated iteratively until feature values converged towards the target class, as given in lines 6–17. In each iteration, firstly, the minimum inter-sample distance, which refers to the distance between data instances of the training set, was computed, as shown in lines 7–15. Afterwards, the calculated distance denoted as step size along with the model and features of a data instance were utilised for explanation vector recalculation, as provided in line 16. If the explanation vector converged toward the target class, then the feature vector of instances to generate the output of potential factors influencing the academic performance of that specific instance, as shown in line 18, was updated. Figure 3C describes the required change to the student features values that changes the prediction to a predefined output (target class or desired performance).

### 3.9 Design and Evaluation of a Dashboard for Data-Driven Feedback and Intelligent Action Recommendations

In this section, we present the process of developing a DFIAR dashboard. The process consisted of four steps: first, the dashboard was designed and developed based on gaps found in the literature review. Second, an expert evaluation was conducted to obtain feedback on the designed dashboard. Third, based on the feedback obtained from the expert evaluation, the dashboard was redesigned and redeveloped. Last, an improved version of the dashboard was evaluated in a real educational setting to examine the utility and limitations of the dashboard for data-driven feedback and action recommendations. In the following sections, both evaluation phases are discussed in detail.

#### 3.9.1 Expert Evaluation

After designing and developing the DFIAR dashboard, the next phase was for the experts to evaluate the dashboard in a workshop setting. The main objective of the workshop evaluation was to gain feedback from domain experts and future users to improve the dashboard before implementing it in a real educational setting. The workshop was conducted in March 2021 at Stockholm university over Zoom.

Participants. Dashboards were presented to 46 participants in a workshop (26 university students and 20 university teachers). The average age of participants was 29 years. The majority of participants (*N* = 43) were experts in the computer science domain and were teaching several computer science courses at different universities. In terms of students, more than half of the students were enrolled in a PhD programme (78%), and 22% were studying in a master’s programme. Regarding teachers, most of the teachers were teaching master’s programmes (90%) and 10% were teaching bachelor’s programmes as well. However, all participants had at least 2 years of experience with prototype and software development.

Procedure. A researcher from our research group presented a 20-min demo about the dashboard, showing the utilisation of different sections of the dashboard and highlighting the specific features of each section. During the demo, log data of students were utilised to demonstrate the dashboard features and participants were allowed to ask questions about each feature. At the end of the workshop, an unmoderated discussion was conducted among the participants to identify the utility and limitations of the dashboard. The reason behind the use of an unmoderated discussion was that we wanted to hear the feedback from experts and future dashboard users. The entire workshop was voice recorded, and during the workshop, our team members also took observational notes.

Data Analysis. The workshop discussion session was transcribed, and the text obtained was merged with observational notes. After that, thematic analysis of the entire text was performed to identify the main discussion topics (themes). Topics were derived from the discussion feedback and organised with respect to the question topics. Lastly, participants’ comments were organised topic wise. The dashboard was then redesigned based on the comments received.

#### 3.9.2 Evaluation in a Real Educational Setting

After the workshop evaluation and redesign of the dashboard, the dashboard was tested in a real educational setting, namely a university distance course. The purpose of this evaluation was to analyse the benefits and challenges that students perceived to be associated with the use of the developed dashboard. The information about the course is presented in *Data Collection*, and information for each module is presented in 3.3.

Participants. We recruited 56 students from a programming course offered in 2021 at Stockholm University in Sweden. The dashboards were available to these students between the 4th and12th week of a 12-weeks course. Over half of the students (61%) used the dashboard at least once (*n* = 34) and 43% (*n* = 24) of those students had continuous interaction with the dashboard throughout the course. Students were from different educational backgrounds such as social science, mathematics, computer science, and electrical engineering. Sixty-eight per cent of users were female, and 32% were male students.

Procedure. At the beginning of the course, a usage guide was developed and presented to the students. In this textual guide, each part of the dashboard, including its features, were presented with examples, for instance, regarding how to log in and how to follow a recommendation. The dashboard access link was available at the top of the course page at Moodle. Students were informed that their dashboard use would not be visible or reported to their course teachers, and consent to collect their usage data was received. The dashboards were turned on after the demo was presented and were turned off at the end of the course. Students’ interactions with the dashboard were logged. At the end of the course, participants were asked for semi-structured interviews, and eight students willingly participated in the interview. To the remaining 26 participants, an open-ended survey was sent, in which the questions were the same as those we asked in the interview, and all participants filled the survey. Questions in interviews and surveys were about the utility and limitations of the dashboard.

Data Analysis. In the interviews and the survey, questions tied to three themes were posed, related to the benefits and challenges associated with data-driven feedback provision, action recommendations and the design of the dashboard. A thematic analysis was conducted to categorise the students’ responses, which included coding, categorisation and thematisation of the interview and survey material. Accordingly, the identified themes were later categorised into three general categories: 1) positive comment—expressing a positive opinion without any concerns; 2) negative comment—expressing either negative observations or some concerns and 3) suggestion—expressing a suggestion/recommendation for future development.

## 4 Results

In this section, we present the experimental results and evaluate the effectiveness of our proposed approach. We evaluate the proposed approach in terms of its ability to predict student academic performance at the assignment level and its ability to identify the influencing factors that affect student performance accurately. Moreover, we present the student dashboard and evaluation results in which we examine the utility and limitations of the dashboard.

### 4.1 Student Academic Performance Prediction Results

In academic performance prediction, our goal was to divide the entire course into three modules, as shown in Figure 2, to predict student performance in the second and third modules in terms of low achievers or high achievers. To achieve this goal, at the end of the first module, student performance on the second module assignment was predicted using the first module data. At the end of the second module, student performance on the third module quiz was predicted using the first and second module data. In the following sections, the results of both predictions are presented.

#### 4.1.1 Prediction of Second Module Assignment Performance

The predictive performance of students on the second module assignment based on the first module data is presented in Table 2. After data resampling, the total number of students increased from 157 to 190, where each class (low achievers and high achievers) consisted of 95 students. According to the obtained results, RF outperformed other ML algorithms in terms of all evaluation measures, with 0.75 accuracy, precision, recall and F-measure, and the value of the area under PRC was high at 0.81. On the other hand, the lowest performance of 0.65 accuracy, precision, recall and F-measure was provided by the MLP. In terms of similarity in results, BayesNet, KNN and SVM gave similar results; however, the PRC area with BayesNet was higher than other ML algorithms.

**TABLE 2:** Comparison of predictive models for performance prediction of student’s second module assignments.

Predictive model	Accuracy	Precision	Recall	F-measure	PRC area
RF	0.75	0.75	0.75	0.75	0.81
MLP	0.65	0.65	0.65	0.65	0.68
BayesNet	0.71	0.71	0.71	0.71	0.76
KNN	0.71	0.71	0.71	0.70	0.63
SVM	0.71	0.71	0.71	0.71	0.62
LR	0.68	0.67	0.68	0.68	0.70

Another set of experiments was conducted to determine the best feature type combination by comparatively analysing all possible feature types. According to the results presented in Table 3, the variety of all feature types provided the highest precision of 0.75 using RF. In contrast, the removal of the initial assessment feature type slightly decreased the precision by 0.01, as shown in a combination of quiz 1, activity completion, and active participation in LMS feature types. However, when activity completion and active participation in the LMS were removed instead of initial assessment, the precision was further reduced to 0.7. Hence, a conclusion can be drawn from these experiments that activity completion and active participation in LMS feature types are significantly correlated with student academic performance.

**TABLE 3:** Feature types of a combination comparison for performance prediction of students’ second module quizzes.

Feature type combination	Predictive model	Accuracy	Precision	Recall	F-measure	PRC area
Initial Assessment + Quiz 1 + Activity Completion + Active participation in LMS	RF	0.75	0.75	0.75	0.75	0.81
Quiz 1 + Activity Completion + Active participation in LMS	RF	0.74	0.74	0.74	0.74	0.79
Initial Assessment + Quiz 1 + Active participation in LMS	RF	0.70	0.69	0.70	0.69	0.77
Initial Assessment + Quiz 1 + Activity Completion	BayesNet	0.68	0.68	0.68	0.68	0.71

#### 4.1.2 Third Module Quiz Prediction

The predictive performance of students in the third module quiz based on both first and second module data is presented in Table 4. After data resampling, the total number of students increased from 157 to 182, where each class (low achievers and high achievers) consisted of 91 students. The results showed that RF outperformed other ML algorithms in all evaluation measures, with 0.91 accuracy, precision, recall and F-measure metrics. Similar to assignment prediction, the value of area under PRC was also notably high at 0.96. Although KNN also presented quite similar results concerning the PRC area, it was 0.11 below RF. On the contrary, the lowest performance of 0.78 accuracy along with 0.78 value for precision, recall, and F-measure was given by LR.

**TABLE 4:** Comparison of predictive models for prediction of students’ performances on third module quizzes.

Predictive model	Accuracy	Precision	Recall	F-measure	PRC area
RF	0.91	0.91	0.91	0.91	0.96
KNN	0.90	0.90	0.90	0.90	0.85
MLP	0.87	0.87	0.87	0.87	0.87
SVM	0.87	0.87	0.87	0.87	0.82
BayesNet	0.85	0.86	0.86	0.86	0.92
LR	0.78	0.78	0.78	0.77	0.80

Similar to assignment experiments, a comparative analysis was conducted to determine the combination of unique feature types among all possible combinations. Table 5 outlines the results of four top-ranked feature type combinations that delivered relatively higher predictive performances. The obtained results demonstrated that the combination of all feature types, except the initial assessment, was best in terms of all evaluation measures. However, by removing activity completion and the initial assessment, the prediction precision was reduced by 0.01, which is equal to the combination with all feature types. Thus, the above results show that the weight of the initial assessment feature type decreased in the middle of the course due to having additional features, such as assignment attributes.

**TABLE 5:** Feature types of a combination comparison for performance prediction of students’ third module quizzes.

Feature type combination	Predictive model	Accuracy	Precision	Recall	F-measure	PRC area
Quiz 1 + Active participation in LMS + Activity completion + Assignment	RF	0.91	0.91	0.91	0.91	0.96
Initial Assessment + Quiz 1 + Active participation in LMS + Activity completion + Assignment	RF	0.90	0.90	0.90	0.89	0.95
Initial Assessment + Quiz 1 + Active participation in LMS + Assignment	KNN	0.90	0.90	0.90	0.9	0.86
Initial Assessment + Quiz 1 + Activity completion + Assignment	KNN	0.89	0.89	0.89	0.89	0.85

### 4.2 Identification of Influencing Factors Through Explainable ML

**FIGURE 4:**
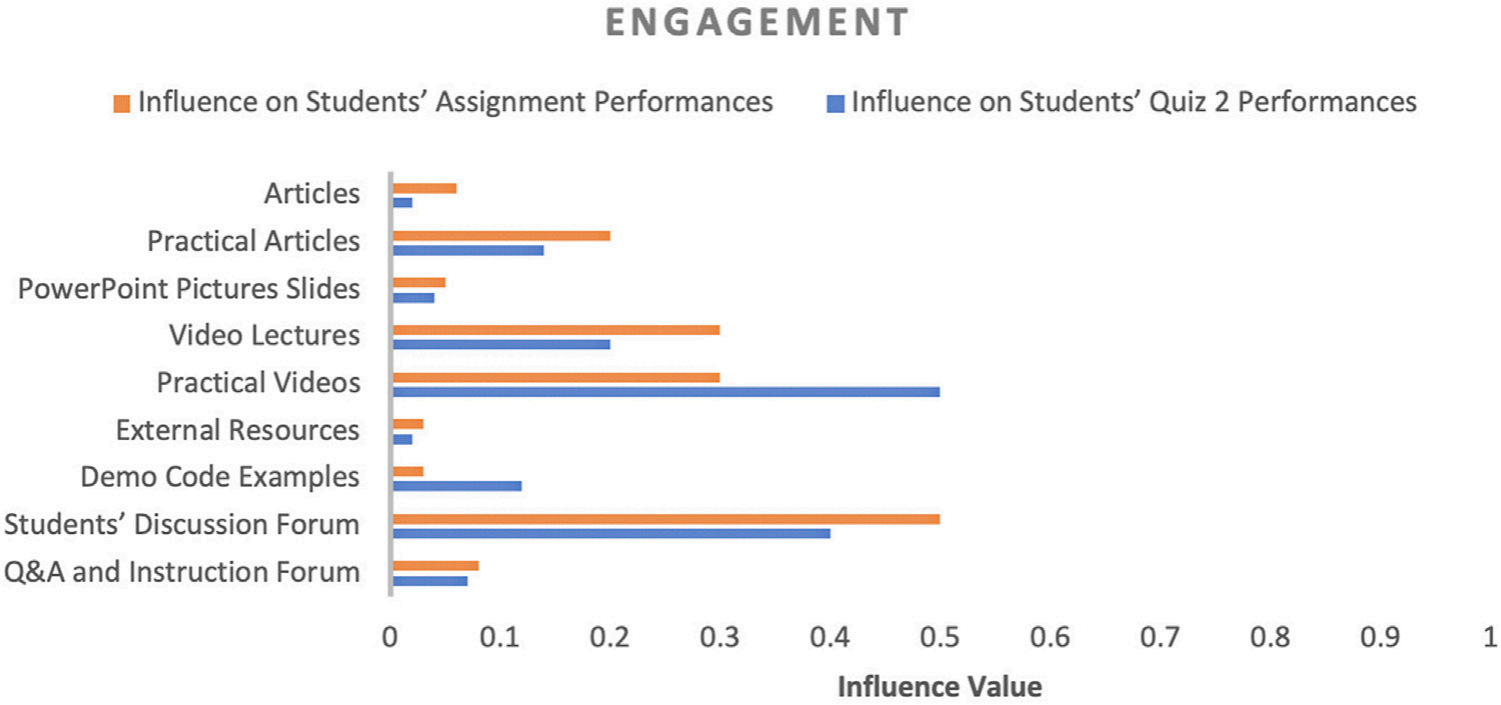
Influence of engagement-related factors on students’ performance.

**FIGURE 5:**
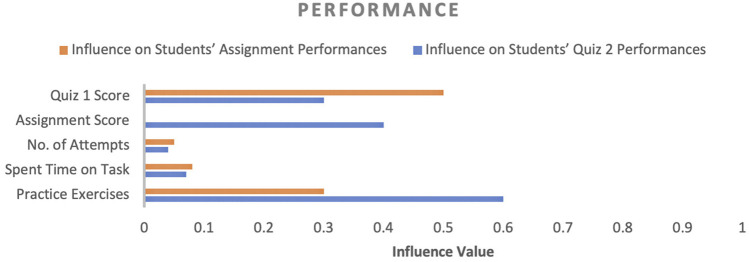
Influence of performance-related factors on students’ performance.

**FIGURE 6:**
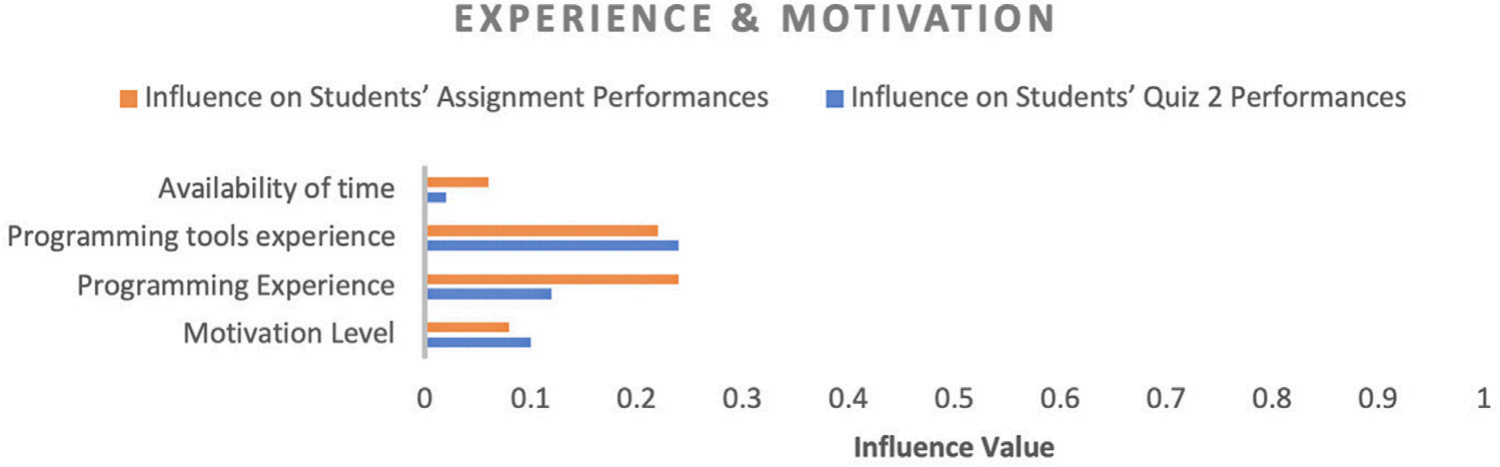
Influence of experience- and motivation-related factors on students’ performance.

**FIGURE 7:**
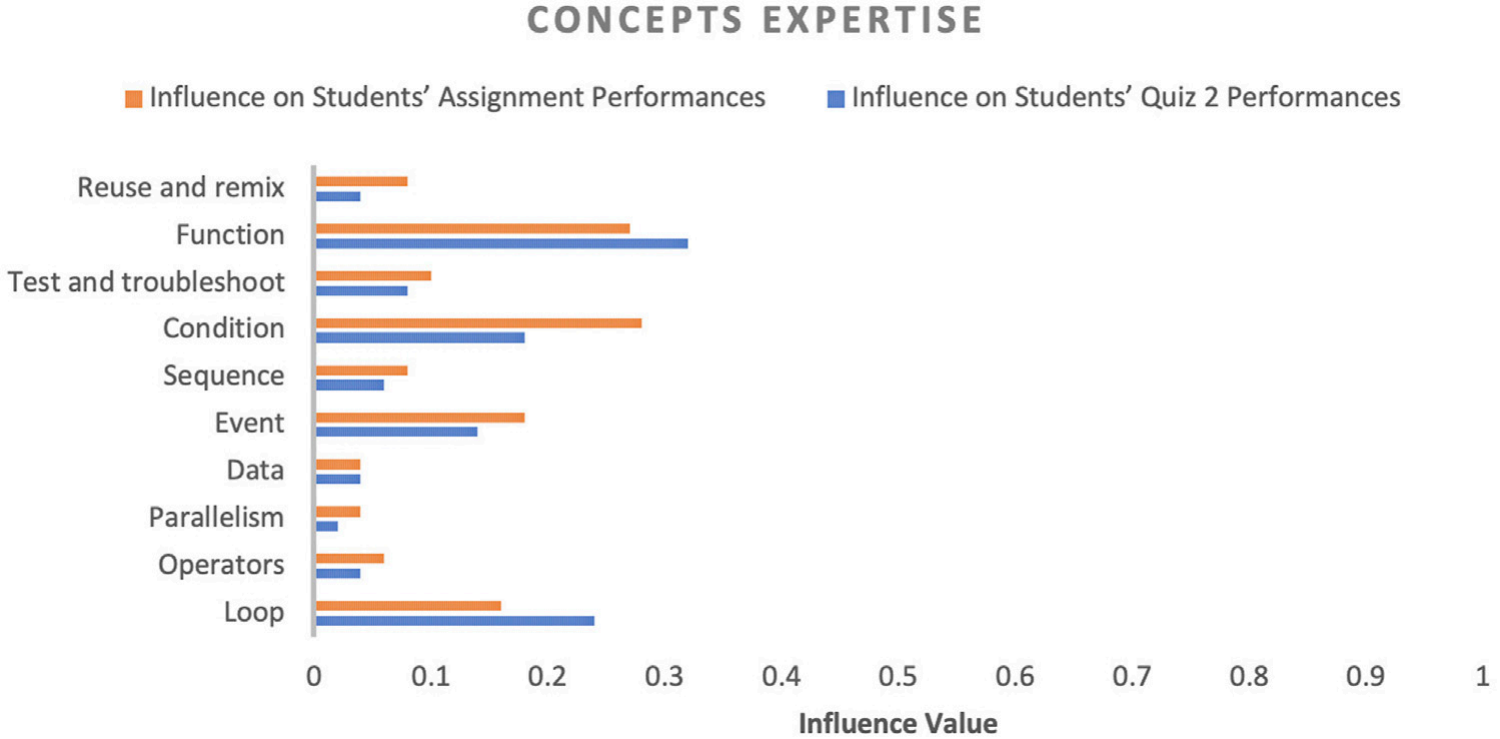
Influence of concept expertise (students’ knowledge of a particular concept) on students’ performance.

In factor identification, our goal was to identify the influencing factors that affect student academic performance and their corresponding influence values. The influence value of a factor designates the impact of the respective factor on student academic performance. Initially, several factors were observed throughout the course and then these factors were categorised into four categories (engagement, performance, experience and motivation, and concept expertise) to convey the purpose of each factor in the course, as shown in Figures 4–7. In the following sections, we discuss the factors of each category in detail.

#### 4.2.1 Engagement

In terms of engagement, practical videos, practical articles (documents containing practical demonstration of course concepts) and students’ discussion forums had a dominant impact (from 0.14 to 0.50) on students’ performance, while external resources, articles (documents containing theoretical explanation of course concepts), and PowerPoint slides had the least impact (from 0.02 to 0.06). Based on both assessment activities, as shown in Figure 4, except for two factors (practical videos and demo code examples), all factors influenced students’ assignment performance more than quiz two because quiz two had several questions for which students were required to study the coding examples and answer the provided code examples. If we compare lecture and practical videos, students’ performance was influenced more by practical videos, although lecture videos were the only source of engagement between the teacher and students. Based on these results, it is evident that in distance programming courses, students’ discussion forums, practical videos and practical articles significantly impact students’ performance more than other sources of engagement.

#### 4.2.2 Performance

Regarding performance, quiz one score and practice exercises (PE) influenced both assessments activities significantly (about 0.5 for quiz 1 and 0.6 for PE); however, when the assignment score feature was added for quiz 2 performance prediction, the quiz 1 score influence decreased. On the other hand, the impact of the PE factor increased (about 0.60 influence score) because quiz 2 required more programming knowledge, which could acquire by performing PE, while the number of attempts and time spent on a task were the least effective, with a maximum impact of 0.05 and 0.08, respectively. The reason to include quiz 1 and assignment score in the factors list is that students were given multiple opportunities to perform both activities. Therefore, if these factors were essential to gain high performance in upcoming assessment activities, teachers could encourage students to focus on these factors more.

#### 4.2.3 Experience and Motivation

Concerning experience and motivation, experience with programming and tools were the dominant factors, and they had almost the same impact on assignment performance, but in the case of quiz 2, the programming experience factor impact decreased drastically (around 50%). Learning material (articles and videos) could be the reason behind this decrease because students had gained enough programming experience through learning material before attempting quiz 2. Motivation was another critical factor; however, its influence value was not as high as the other factors, but it still played a vital role in close predictions where predicted performance and actual performance did not have much difference.

#### 4.2.4 Concept Expertise

In terms of concept expertise, this study found that expertise in function, condition, event and loop concepts were highly impactful on both assessment and activity performance. At the same time, expertise in data, operators and other factors were not very impactful. Let’s analyse concepts’ impact on individual assessment activities. We could visualise that each factor’s influence value was high on assignment performance compared to the quiz two assessment activity because as the course progressed and students utilised course materials, they acquired an understanding of basic concepts. However, a couple of concepts, such as function and loop influence, increased because these concepts are complex. Obtaining expertise in both concepts required small assignments in which students could evaluate their knowledge of these concepts.

### 4.3 Dashboard for Intelligent Action Recommendations

**FIGURE 8:**
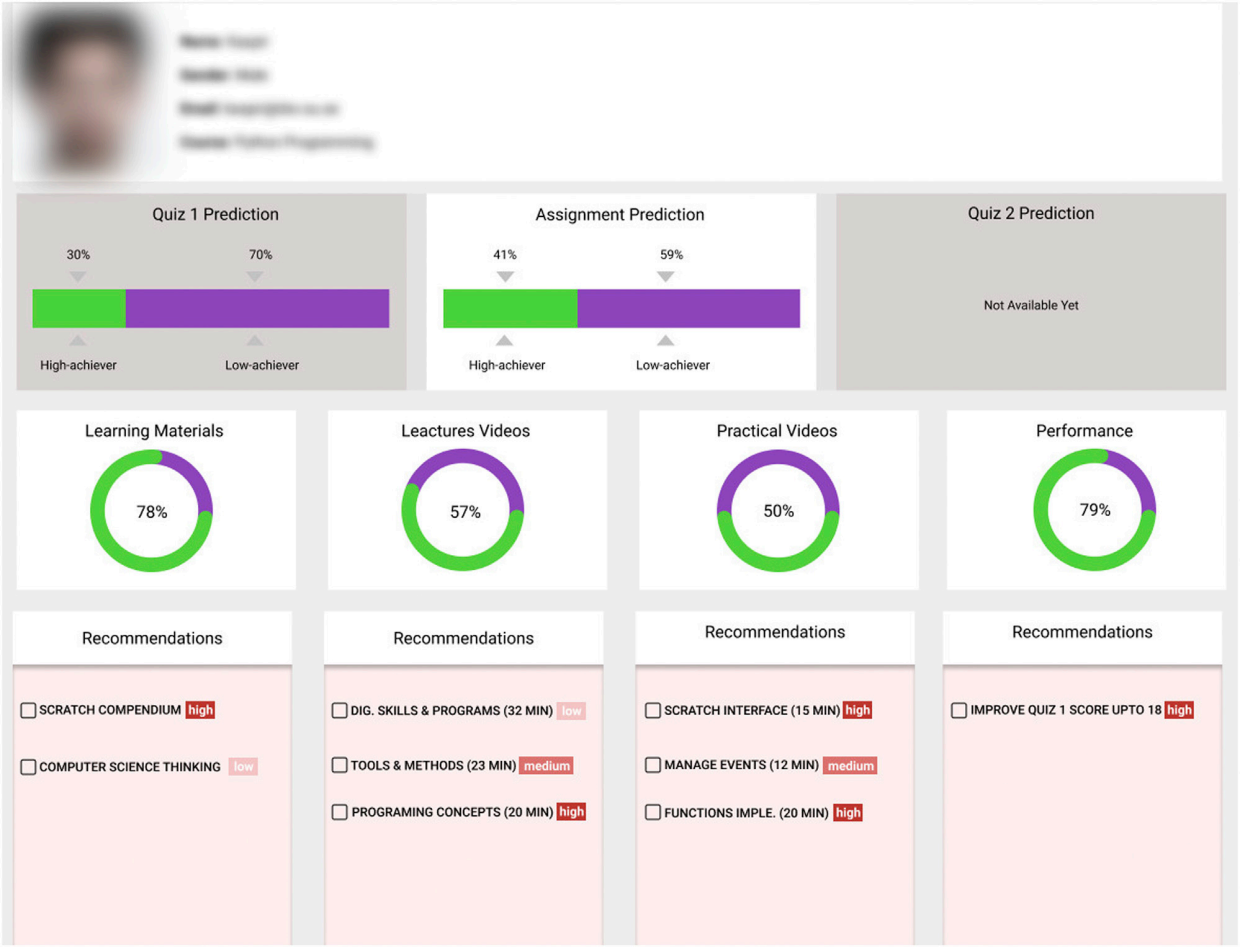
Student dashboard.

The proposed explainable ML-based algorithm’s output is the list of factors, their influence values and required changes; however, it is still complicated for students to understand and interpret such information. Therefore, to generate more informative feedback along with actionable recommendations, dashboards were developed for students. The student dashboard, as depicted in Figure 8, consists of three main components. 1) The prediction component offers the predicted performance probability for each assessment activity (quiz or assignment) in terms of low and high achievers. For example, the assignment prediction box shows that this particular student would perform as a low achiever in the following assessment activity, an assignment, with a 59% probability. At the same time, there is a 41% probability that this student would be a high achiever. However, why this student would be a low achiever is not provided in the prediction component. Therefore, we introduced (2), the progress component, that informs students in which areas they are lacking and how much progress is required. For example, as shown in Figure 2, this student did not watch all the lectures and practical videos necessary to achieve a higher performance, and it was mandatory to watch each of them before submitting the assignment. So, the progress component presents this information as progress circles along with a percentage to show the student their current situation.

However, which videos should be watched and the order are not shown in the progress component. Hence, we introduced (3), a recommendation component, that adds a recommendation box under each progress circle to inform the student about the list of essential items and their priority level to perform to achieve a high performance. These recommendations stemmed from the factors which were identified using Algorithm 2. The dashboard is delivered to the students at the beginning of the course and it is stopped at the end of course. Meanwhile, real-time students’ interaction data with LMS is collected and processed to provide feedback and action recommendations. Students follow these recommendations successively and their probability to pass the course exam is increased.

### 4.4 Evaluation

This section presents the evaluation results of the DFIAR dashboard in the different evaluation settings to verify the usability and functionality of the dashboard components and to examine the benefits and challenges of the dashboard for students. In the following sections, we first present the evaluation results in the context of the experts’ workshop, and then the evaluation results in a real educational environment are presented.

#### 4.4.1 Results of the Experts’ Workshop Evaluation

The workshop discussion and observational notes detected four main discussion topics, including action recommendations, data utilisation, machine learning approaches and dashboard design. Overall, 46% of the feedback and questions were about action recommendations, 27% were about data utilisation, 17% were about machine learning approaches and the remaining were about design.

With regard to action recommendations, participants discussed the possible advantages and disadvantages of recommendations for students’ academic performance. Overall, 80% (*N* = 37) of participants were positive about the recommendations generated because they believed most of the recommendations were actionable and students could self-regulate if they followed the recommendations during the course. On the other hand, 20% (*N* = 9) of participants raised questions about the harmful effects of the recommendations on students’ studies. Four participants agreed on the need for further examination of the generated recommendations to ensure that the recommendations did not have any negative impact on students.

Regarding data utilisation, nearly 89% (*N* = 41) of participants were satisfied with the utilisation of students’ data. However, eight participants raised questions about the size and types of data because they assumed that recommendations were being generated using a machine learning model; therefore, size and types of data matter and should be diverse. One of the participants mentioned that “*if additional attributes of videos (e.g. time to watch and coverage) had been added, then video-related recommendations could be improved*”. In terms of machine learning approaches, 30% (*N* = 14) of participants suggested the need to test more machine learning algorithms such as fuzzy-based models to improve the overall accuracy. In comparison, the remaining 70% (*N* = 32) provided positive feedback on the utilisation of machine learning algorithms, and they were content with the existing prediction accuracy. Regarding design, almost all participants agreed that the dashboard “*design is simple, well understandable and easy to use*”. However, one participant commented that “*An inquiry should appear when a student follows one of the recommendations to make sure that either the student completed that recommendation or not. For example, when a student watches a lecture video, then a question should be asked whether he/she watched the entire video and whether that particular video improved his/her knowledge or not*”.

#### 4.4.2 Results of the Evaluation in a Real Educational Setting

The percentages for participants’ responses based on the coding scheme described in section 3.9.2 are outlined in Table 6. As shown in Table 6, in terms of feedback, overall 82% (*N* = 28) of participants provided positive responses, where 22 out of 28 respondents were highly satisfied with the feedback provided in light of the student prediction outcomes and their level of understanding. Several respondents summarised the experience as follows: “*The feedback was well organised, and it showed a clear picture of how much I have left to do*”. Similarly, another respondent echoed the positive view by saying, “*It was useful for me in this sense that it somehow accelerated my studies by providing visuals of my progress*”. Another respondent asserted: “*The tool gave me the information that I had missed in one of the lectures, which was good to know before the assessment. I think it will be highly motivational and helpful for bigger courses*”. On the negative side, 18% (*N* = 6) of respondents raised questions about the accuracy of prediction outcomes. One participant responded: “*I got 18 scores on the quiz assessment, but feedback showed me I will get 12 scores*”. Another responded expressed: “*The provided dashboard feedback really did not work for me*”, without explaining further. We received a number of suggestions from the participants regarding how to improve feedback. A huge number of participants suggested that “*feedback notifications should be sent every day about their progress to keep them updated*”.

**TABLE 6:** Summary of participants’ responses from real educational setting evaluation.

Question themes	Response category	% of responses	Response scaling	
			High	Low
Feedback	Positive	82 (%)	80 (%)	20 (%)
	Negative	19 (%)	13 (%)	87 (%)
	Suggestion	23 (%)	60 (%)	40 (%)
Recommendation	Positive	85 (%)	91 (%)	9 (%)
	Negative	15 (%)	19 (%)	81 (%)
	Suggestion	19 (%)	70 (%)	30 (%)
Design	Positive	91 (%)	93 (%)	7 (%)
	Negative	9 (%)	6 (%)	94 (%)
	Suggestion	18 (%)	12 (%)	88 (%)

With regard to recommendations, 85% (*N* = 29) of participants felt motivated by the recommendations they received, and 20 out of 29 respondents viewed the action recommendations as beneficial, stating, for instance, that “*Due to the complex course structure, the course recommendations helped me to regulate myself and improve my learning and performance*”. On the other hand, several respondents shared the positive comment by saying, “*Yes, provided recommendations proved helpful to improve the course grades*”. Similarly, a couple of respondents stated: “*The provision of low and high priority in recommendations was a good sign while choosing recommendations, and it also assisted*”. On the negative side, 15% (*N* = 5) of respondents were not pleased with the recommendations provided. One participant clarified this with the following: “*I am not satisfied, as most of the recommendations were videos and less were practical exercises*”. Another respondent clearly stated: “*I did not check the recommendations because I do not think that these will help me*”. Two participants provided suggestions along with positive reactions, as one of the respondents reported: “*I found the dashboard useful from a recommendations perspective, but it still needs improvement, as I once observed that some tasks that I have already attempted were still in the recommendations list*”.

With respect to design and interface, overall 91% (N = 31) of participants claimed that the dashboard interface was understandable and easy to use, as 22 out of 31 did not want any improvements to the interface. Eight respondents commented by saying, “*This tool is very easy to use, as its Graphical User Interface (GUI) is easy to follow*”. In the same context, three respondents mentioned: “*The visuals of the progress bar and pie charts made this tool easy to use*”. On the negative side, 9% (N = 3) of respondents were confused by the dashboard interface. One participant responded: “*At the beginning I did not know how to follow the recommendations and what the meaning was of priority*”. Another student emphasised that “*It was difficult to understand this interface on my mobile gadget*”. We received a few suggestions related to the design of the dashboard interface, such as many participants suggested that the percentages provided seemed ambiguous and they needed more clarification by drawing a GUI that distinguishes between the percentage performance and the percentage study. A couple of respondents offered suggestions along with positive responses; one said: “*It is something great but may be nicer if they put a small “question mark” icon on charts that give detailed information about them*”.

**Statistical Analysis:** Two statistical analyses were performed to examine the dashboard effectiveness: the first analysis was conducted to find the correlation between followed recommendations and students’ performance. The second analysis was conducted to find out the significance of dashboard utilization on student academic performance.

**FIGURE 9:**
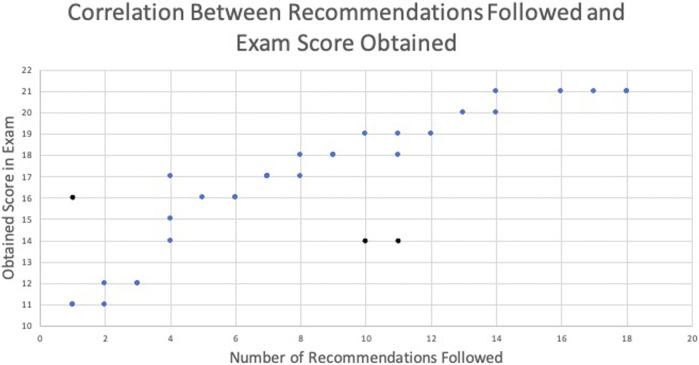
Correlation between recommendations followed and students’ scores.

To determine whether the following recommendations (FR) were correlated with students’ performance, on the last course examination (CE), Pearson’s correlation coefficient (Sedgwick 2012) was calculated based on the dashboard usage data. The calculation outcome showed a positive correlation between FR and CE with r (34) = 0.86, *p* < 0.0001. Graphically, this is illustrated in Figure 9, which presents a plot of the number of recommendations followed along the *x*-axis versus the examination score obtained along the *y*-axis. According to the information provided by this plot, the overall score obtained by students increased as the number of recommendations followed increased. To determine the effectiveness of the dashboard on students’ academic performance, another statistical analysis, a *t*-test (T. K. Kim 2015), was conducted for two groups of students (20 students in each group) independently. Group 1 consists of students who utilized the dashboard between the assessment activities (Quiz1 and Quiz2, as shown in Figure 2). Group 2 consists of students who did not use the dashboard between assessment activities. The inclusion criteria for both groups were that the students were randomly selected and they had completed all course assessments. In this analysis, Quiz1 and Quiz two scores of students in each group were utilized to perform a *t*-test. The obtained results in terms of t-value and *p*-value showed a significant statistical difference (t = 6.688, *p* = 0.0001) in Group 1 students’ performance while Group 2 students’ difference was not significant (t = 2.026, *p* = 0.055). As shown in both statistical analyses, the dashboard positively impacted students’ learning and helped them reach the desired academic performance.

## 5 Discussion

The purpose of this study was to propose an explainable ML-based approach in LA to predict student performance at an assessment level and to identify which factors affect student performance. We also aimed to implement and evaluate the proposed approach as a DFIAR dashboard to examine its utility and limitations for students. We discuss our results and the implications of these findings in the following sections.

### 5.1 Findings and Implications

The first aim was to explore how student performance could be accurately predicted at the assignment or quiz level, using established ML algorithms. We found that random forest-based predictive models provided satisfying results when utilising the education data of students taking online programming courses. Since prediction is entirely dependent on the data supplied, we attempted to identify the association between data variables and academic achievement. We found that active participation in an LMS, quiz score and assignment grade attributes were essential to determining student academic performance because they were positively correlated with student performance throughout the course. However, the importance of the initial assessment attributes decreased during the middle of the course due to the additional attributes, such as practical videos and assignments. One of the practical implications of these findings is that prediction at the assessment level increases students’ interest in learning and motivates them to adapt the course material and learning paths to meet their needs better. Such predictions could also support teachers in engaging in timely interventions to help students who are predicted to achieve low results.

These prediction findings led us to the first research question: Can we employ explainable ML approaches to identify factors that affect students’ academic performance at the assignment or quiz level in order to provide automatic and data-driven feedback? As illustrated in the results section, we found that explainable ML was not only able to identify the potentially influencing factors from courses currently running, but it was also able to identify the necessary change in the feature value to reach high performance. Moreover, we found five factors, such as active participation in the LMS, forum discussion, programming experience, quiz score and assignment grade, that dominated students’ academic performance. The required value change in influencing factors provided an additional edge to the proposed approach against existing approaches to factor identification (Akhtar et al., 2017; Lu et al., 2018; Ahuja et al., 2019; Bibi et al., 2019; Kamal and Ahuja 2019), which were also able to identify the factors but were not able to calculate the required value change to reach a high performance on an individual level. Since changing the value of one factor could affect the whole prediction, the appropriate estimation of the value change for each prediction helped to calculate the real impact of each factor. The proposed approach utilised the accurate performance prediction and identified factors along with their impact and required change (as shown in Figure 3) to develop a dashboard for the data-driven feedback and action recommendations.

The prediction and factor identification findings led us to the second research question: What is the perceived utility and limitations of the dashboard for data-driven feedback and action recommendations for students? As presented in the dashboard evaluation results section, we found three main benefits. First, the dashboard helped students to self-regulate, especially when the course structure is complex and students have to read and watch extensive course material. In this case, the dashboard assisted students’ regulation of learning by providing action recommendations that highlighted the course resources the students should focus on next in order to increase the probability of improved course performance. Moreover, the outline of recommendations along with the progress bar provided allowed students to monitor their progress and plan adjustments to improve their learning process. The second benefit concerned the level of motivation to learn by providing data-driven feedback in terms of estimated assessment scores. The provision of assessment scores after following a recommendation was found to be an interesting aspect of the dashboard that motivated students and increased their interest in following more recommendations. Another motivational aspect, which was revealed from the results regarding the dashboard interface and was found to be easy to use and interpretable by students, was the dashboard’s friendly interface. This also motivated students to engage in their studies, leading to enhanced learning outcomes. Yet another identified benefit was related to the impact generated when following action recommendations. Although the sample of students was relatively small in this study, a significant correlation was found between the number of recommendations followed and students’ final course performance.

In terms of limitations, the dashboard is unable to provide evidence about whether the recommendations followed improved student knowledge about course concepts or not. For example, after following a recommendation, the dashboard does not provide any mechanism, such as a questionnaire, to students so that they can verify that knowledge has been constructed/assimilated based on the recommendation followed. Moreover, the dashboard interface is limited to providing a predictive performance of the students, as it does not display the highest scores in the course and does not allow students to compare their current learning with that of their peers.

These findings are interesting from four perspectives. From the student’s perspective, a potential practical implication of the findings of this work is that the students improve their learning outcomes by self-monitoring of their progress. It leads them to complete their courses and achieve the desired educational goals. From the perspective of teachers, these findings are helpful to save teacher time by providing students with a self-regulatory environment that aware students of their learning status and guide them regarding suitable learning material individually. This assistance to teachers reduces their workload and helps to improve their other pedagogical practices, such as enhancing course structure content and course-related activities. From the institutions perspective, these findings are helpful to improve students’ retention rate by providing timely guidance in terms of their predictive assessment scores. It causes that student’s rate of dropping out is declined, and they ultimately can achieve their course degree at the time. However, lack of student’s self-regulation causes many pedagogical and societal issues, such as teachers have to make much effort to deliver appropriate instructions to each student regarding the study content in a course. Similarly, by increasing the dropout rate, on the one hand, most students are failed that causes them to be psychologically demoralized. On the other hand, state money invested in different education programs is wasted.

From a generalizability perspective, we claim that the proposed approach is quite applicable to diverse educational courses. To apply the approach to a new course, all possible modalities of data for a new course, e.g., quizzes, assignments, practical exercises, watched videos, reading material, project tasks, and final exams, are taken as input. Then a feature analysis, including feature extraction and selection, is performed again except on common features such as LMS usage and motivation level. Since features are specific to courses, the ML model is rebuilt and could then predict student academic performance accurately. Afterwards, the LIME framework can be employed on the built ML model and extracted features to identify the influencing factors. Lastly, identified factors can be used to provide intelligent feedback and action recommendations on a dashboard.

### 5.2 Limitations and Future Recommendations

In terms of limitations, this paper performs experiments on a programming course that limits its generalizability, but we see that generalizability is not negatively affected due to two reasons. First, model building in the proposed approach was on a very general across subject didactical structure because the participants were from different disciplines such as mathematics, social sciences, and electrical engineering. Second, the developed recommender dashboard can be used in diverse online courses as it was based on a very specific way of designing an online course that consisted of all imaginable online course components such as videos, audios, assignments, and quizzes etc. and allows the students to follow them in a chronological temporal order. The second main limitation is about sample size for experimentation. The experimental set for evaluation of the recommender system consisted of only 34 participants. However, in the future, we plan to perform an evaluation at a large scale with big sample size.

In our future work, we will emphasize how to improve the utility of the dashboard by adding more functionality and easier to use design components in its interface. Furthermore, the confronted challenges and limitations such as generalizability will also be stressed to work on in the future prospect. One other future direction is to make a dashboard for teachers that could be useful in pedagogical practices. In this dashboard, recommendations will be provided to teachers while providing helpful instructions in their study. Moreover, the used LIME framework provides a local explanation or local influences of features that give importance to features in a particular observation instead of providing a global influence in which the overall importance of features for the predictive model is provided. Since this drawback of the LIME framework affects the quality of data-driven feedback and action recommendations provided on the dashboard, one of our future directions will be the provision of the global influence of features to improve recommendations for actions. Furthermore, data anonymization is among our future directions so that the data processing steps can enter the scrutiny of a public dataset.

## 6 Conclusion

In this paper, we have proposed an explainable ML-based approach to provide automatic and intelligent effective feedback and action recommendations for students. In this approach, an explainable ML-based algorithm has been developed that utilises students’ LMS data and builds predictive models to compute data-driven feedback and recommendations for action at the assignment or quiz level, which can help students to self-regulate their learning and improve their academic performance. To examine the effectiveness of the proposed approach, a dashboard was designed, developed and implemented in a real course setting. Evaluation of the implemented dashboard was conducted by identifying the utility and benefits of the dashboard. Evaluation results showed that the dashboard helped students in self-regulation, boosting their motivation and improving their academic performance. In the future, we will perform experiments on larger data sets to ensure that the proposed approach is sustainable, and we will not need to resample the data to avoid data imbalance problem. Moreover, we will collaborate with course teachers to deploy and evaluate our dashboards in massive courses to determine their efficacy.

## Data Availability

The datasets presented in this article are not readily available because Dataset was collected to improve university students' performance and for course improvements. Therefore, it will not available to the public. Requests to access the datasets should be directed to muhamamd.afzaal@dsv.su.se.
